# Gamma Oscillations in the Temporal Pole in Response to Eyes

**DOI:** 10.1371/journal.pone.0162039

**Published:** 2016-08-29

**Authors:** Wataru Sato, Takanori Kochiyama, Shota Uono, Kazumi Matsuda, Keiko Usui, Naotaka Usui, Yushi Inoue, Motomi Toichi

**Affiliations:** 1 Department of Neurodevelopmental Psychiatry, Habilitation and Rehabilitation, Graduate School of Medicine, Kyoto University, 53 Shogoin-Kawaharacho, Sakyo-ku, Kyoto, 606–8507, Japan; 2 Brain Activity Imaging Center, Advanced Telecommunications Research Institute International, 2-2-2 Hikaridai, Seika-cho, Soraku-gun, Kyoto, 619–0288, Japan; 3 National Epilepsy Center, Shizuoka Institute of Epilepsy and Neurological Disorders, Urushiyama 886, Shizuoka, 420–8688, Japan; 4 Faculty of Human Health Science, Graduate School of Medicine, Kyoto University, 53 Shogoin-Kawaharacho, Sakyo-ku, Kyoto, 606–8507, Japan; Universitatsklinikum Tubingen, GERMANY

## Abstract

The eyes of an individual act as an indispensable communication medium during human social interactions. Functional neuroimaging studies have revealed that several brain regions are activated in response to eyes and eye gaze direction changes. However, it remains unclear whether the temporal pole is one of these regions. Furthermore, if the temporal pole is activated by these stimuli, the timing and manner in which it is activated also remain unclear. To investigate these issues, we analyzed intracranial electroencephalographic data from the temporal pole that were obtained during the presentation of eyes and mosaics in averted or straight directions and their directional changes. Time–frequency statistical parametric mapping analyses revealed that the bilateral temporal poles exhibited greater gamma-band activation beginning at 215 ms in response to eyes compared with mosaics, irrespective of the direction. Additionally, the right temporal pole showed greater gamma-band activation beginning at 197 ms in response to directional changes of the eyes compared with mosaics. These results suggest that gamma-band oscillations in the temporal pole were involved in the processing of the presence of eyes and changes in eye gaze direction at a relatively late temporal stage compared with the posterior cortices.

## Introduction

It has been said that the eyes are windows to the soul [1] and thus are indispensable for human communication. Accordingly, the detection of eyes and the recognition of changes in eye gaze direction generate multiple psychological activities in the observer. For example, straight and averted eye directions trigger emotional reactions [2] and attention orienting [3], respectively, and both induce mind reading [4]. These processes play important roles in real-life social interactions [5] as well as contribute to impairments in social functioning in patients with clinical disorders [6].

Neuroscientific studies have explored the neural mechanisms underlying the processing of eye information and have identified several brain regions that are activated in response to eyes and eye gaze direction changes. Several neuroimaging studies using functional magnetic resonance imaging (fMRI) and positron emission tomography (PET) demonstrated that the posterior superior temporal sulcus (STS) and the adjacent middle and superior temporal gyri (STS region [7]) are active in response to the presence of eyes and changes in eye gaze direction, specifically in the case of averted gaze direction [8–16]. Intracranial electroencephalography (EEG) studies also reported that the STS region [17] and the inferior occipital gyrus (Sato et al., submitted) exhibit electrical activity in response to eye gaze direction changes and eyes, respectively. Additionally, neuroimaging studies revealed that other anterior regions, such as the amygdala, are also active in response to eyes and eye gaze direction changes [16,18]. Because these regions are also more active during the presentation of faces relative to non-face objects (for a review, see [19]), it is likely that the processing of eyes is accomplished as a subtype of processing for faces. These findings suggest that eye information is processed across a widespread neural network that includes the inferior occipital gyrus, STS region, and amygdala.

However, it remains unclear whether there are changes in temporal pole activity in response to the presence of eyes and changes in eye gaze direction. Lesion studies in monkeys have revealed that the temporal pole plays an indispensable role during social interactions [20–23]. These findings are consistent with those of comparative anatomical studies indicating that the temporal pole exists only in primates [24,25], likely because primates are exceedingly social animals. Anatomical studies in monkeys revealed that the temporal pole receives highly processed visual signals from the posterior cortices, including the STS region, and communicates bidirectionally with the amygdala (for a review, see [26]). This suggests that the temporal pole may cooperate with these regions during the processing of eye information. Several previous neuroimaging studies in humans reported that the temporal pole is more activated in response to faces relative to other objects [27–30], suggesting that this region is involved in face processing. Although none of the aforementioned neuroimaging studies observed an activation of the temporal pole in response to eyes or eye gaze direction changes, this null finding might be accounted for by air-tissue inhomogeneity artifacts that can diminish fMRI signals in this region or by ill-defined anatomical categories (e.g., the periamygdaloid region) due to the lack of theoretical interest [31]. On the other hand, an fMRI study showed activation in the temporal pole by comparing brain activation during the observation of eyes in a mind reading task versus during the passive observation of crosshairs [32]. Another fMRI study investigated brain activation changes during the adaptation to eye gaze direction and reported reduced activity in the temporal pole in response to adapted gaze direction [33]. Based on these findings, we hypothesized that direct recording of electrical activity from the temporal pole could reveal its activation in response to the presence of eyes and changes in eye gaze direction.

Furthermore, if the above hypothesis is correct, the timing and manner in which the temporal pole is activated during the processing of eye information need to be addressed. To understand the neural mechanisms, that is, the causal relationships among brain regions, the time course and frequency of brain activation should be evaluated [34,35]. To date, no electrophysiological studies, which can provide this type of information, have investigated activation in the temporal pole in response to the presence of eyes and changes in eye gaze direction. Indirect evidence from intracranial EEG recording and its event-related potential (ERP) analyses revealed temporal differences in the activation of the temporal pole and other visual areas during face processing [36]. This study showed that the temporal pole exhibits a higher ERP component that peaks at 300–400 ms in response to faces than to other objects, whereas other posterior cortices, including the inferior occipital gyrus and STS region, exhibit the ERP peaks at approximately 200 ms. Other intracranial EEG studies investigating the processing of eye information revealed that the inferior occipital gyrus (Sato et al., submitted) and STS region [17] exhibit ERPs that peak at approximately 200 ms. Collectively, these findings suggest that the temporal pole may exhibit ERPs related to eye processing during the 300–400 ms window that have a similar temporal profile to those exhibited during face processing. However, it must be noted that ERP analyses primarily detect the low-frequency components of EEG data [37]. For the comprehensive elucidation of high- and low-frequency neuronal activities at a high temporal resolution, time–frequency analyses must be conducted [38]. Previous intracranial EEG studies that performed time–frequency analyses revealed that the inferior occipital gyrus (Sato et al., submitted) and STS region [17] show gamma-band (higher than 30 Hz [39]) activation in response to eye information beginning at approximately 100 ms. These data suggest that, within the visual stream from the posterior to the anterior occipito–temporal cortices, the temporal pole may exhibit gamma-band activation during about 200–300 ms, which would be later than that observed in more posterior regions. Taking these findings together, we hypothesized that the temporal pole could show gamma-band and ERP activations beginning at approximately 200–300 and 300–400 ms, respectively, in response to the presence of eyes and changes in eye gaze direction.

To test these hypotheses, we analyzed intracranial EEG data from the temporal poles of six participants undergoing presurgical assessments for epilepsy. To examine the effect of appearance of eyes, the participants were presented with visual stimuli comprising the eye region or mosaic patterns as control stimuli. To test the effect of eye gaze direction, averted and straight directions were prepared for both the eyes and mosaics. To explore the effect of changes in eye gaze direction, a second stimulus was presented 500 ms after the onset of the first stimulus with the direction of the second stimulus being different from that of the first. To examine automatic eye processing, the participants were asked to engage in dummy target detection. Time–frequency statistical parametric mapping (SPM) [40] and traditional ERP analyses were conducted. Because several lines of evidence indicate that there are functional hemispheric differences during the processing of eye information [41], the activities of the temporal poles in both hemispheres were analyzed and compared.

## Materials and Methods

### Ethics Statement

This study was approved by the Ethics Committee of Shizuoka Institute of Epilepsy and Neurological Disorders, and was conducted in accordance with the approved guidelines. All participants gave written informed consent after being provided with an explanation of the experimental procedures.

### Participants

The present study included 6 patients (5 females and 1 male; mean ± *SD* age, 34.5 ± 7.9 years). All participants were suffering from pharmacologically intractable focal epilepsy and underwent the implantation of intracranial electrodes as part of a presurgical evaluation. The experiment was conducted 2.0–2.8 weeks after the electrode implantation. The surgical evaluations suggested that the main epileptic foci for all the participants were outside the temporal pole; five of the foci were in the hippocampus and one was in the lateral temporal cortex.

Neuropsychological assessments confirmed that all participants’ language ability and everyday memory were intact. The intelligence quotient (IQ), measured by the revised Wechsler Adult Intelligence Scale, was in the normal range in five participants, and in the mildly mentally retarded range in one participant (mean ± *SD* full-scale IQ: 91.8 ± 19.2; mean ± *SD* verbal IQ: 86.7 ± 12.0; mean ± *SD* performance IQ: 100.7 ± 27.3). During the experiment, no seizures were observed and all participants were mentally stable. All participants were right-handed, as assessed using the Edinburgh Handedness Inventory [42], and had normal or corrected-to-normal visual acuity. The data from different electrodes are reported elsewhere [43].

### Anatomical magnetic resonance imaging (MRI) assessment

Pre- and post-implantation anatomical assessments were conducted using the 1.5-T structural MRI scanning system (Signa TwinSpeed, General Electric Yokokawa) and multi-slice computed tomography (CT) scanner (Millennium VG, GE Medical Systems). For each participant, a whole brain T1-weighted MR image (matrix size = 256 × 256, field of view = 22 × 22 cm, and 76 slices resulting in voxel dimensions of 0.8594 × 0.8594 × 2.0 mm thick) and CT image (matrix size = 512 × 512, field of view = 22.8 × 22.8 cm, and 24 slices resulting in voxel dimensions of 0.4453 × 0.4453 × 5.0 mm thick) were acquired. Pre-implantation anatomical MRI and CT assessments and surgical evaluations did not reveal any structural abnormalities in the bilateral temporal poles of any participant.

**Fig 1:**
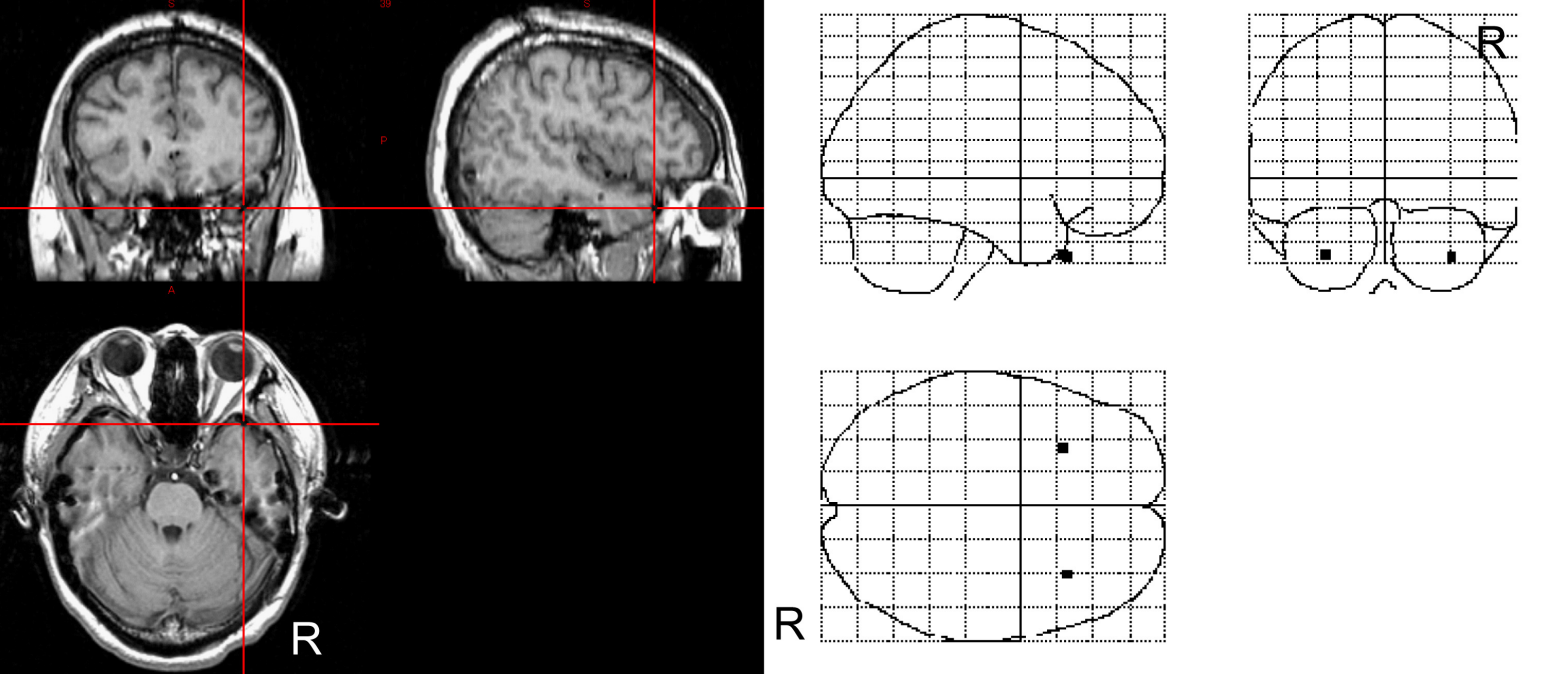
Location of electrodes in the temporal pole. Left) Representative anatomical magnetic resonance images. Cross hairs indicate the electrode location in the temporal pole. Right) Averaged coordinates of the electrodes in the temporal pole in the Montreal Neurological Institute space.

The intracranial electrodes were implanted using the stereotactic method [44], and the implantation sites were chosen based solely on clinical criteria. Subdural electrodes were implanted in the usual manner in both hemispheres for five participants and in the right hemisphere for one participant. Post-implantation anatomical MRI and CT assessments were conducted to confirm the positions of the electrodes of interest in the temporal pole. First, individual MRI data were segmented into gray matter, white matter, cerebrospinal fluid, skull, and scalp using the unified segmentation and normalization procedure [45] in SPM8 (http://www.fil.ion.ucl.ac.uk/spm/) implemented in MATLAB 2012b (MathWorks). Next, individual CT data were thresholded to remove the cranial content of the brain. Note that both the bony skull and the high-intensity electrode signals were preserved in this image processing. The resulting CT skull image was then co-registered to the skull image derived from MRI segmentation. To report the stereotactic coordinates of the electrode positions, individual T1-weighted MR images were normalized to a standard T1 template image defined by the Montreal Neurological Institute (MNI). The spatial transformation parameters from this normalization process were then applied to the gray matter image and the CT skull image. The CT skull image was re-thresholded and then binarized to limit the image contents to electrodes only. Finally, each electrode was well visualized in the brain surface-rendering of the gray matter image using the overlay function in MRICRON software (http://www.mccauslandcenter.sc.edu/mricro/mricron/). One of the study authors manually localized and confirmed the electrodes in the temporal pole. The mean ± SD MNI coordinates of the electrodes were as follows: right, x 33.8 ± 4.4, y 22.7 ± 3.2 z -39.3 ± 4.7; left, x -28.7 ± 5.2, y 21.2 ± 2.5, z -38.3 ± 2.7. The mean three-dimensional locations of the electrodes were projected on the MNI glass brain (SPM maximum intensity projection format; Fig 1).

### Stimuli

**Fig 2:**
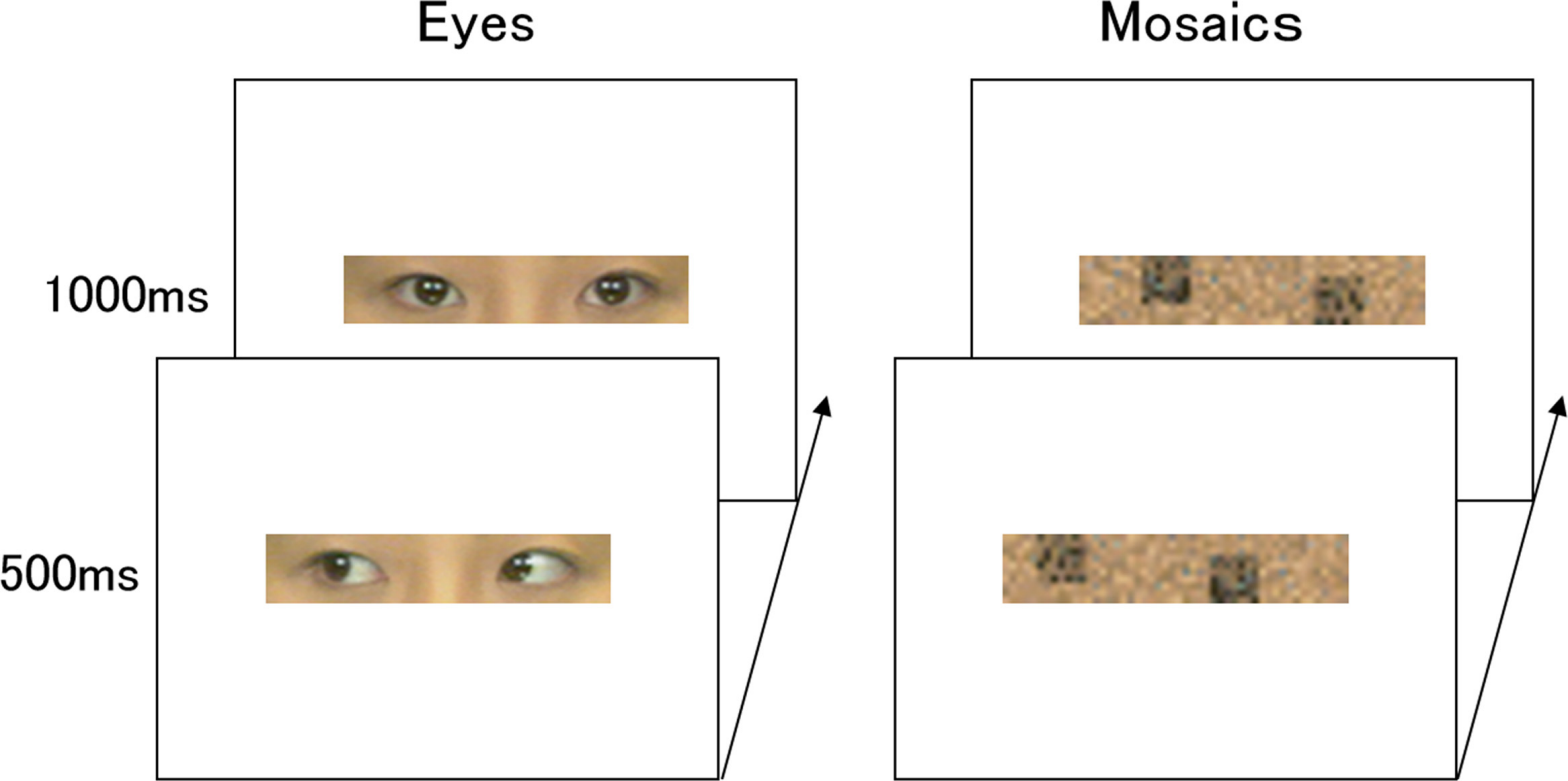
Illustrations of the stimuli. The averted (first presentation)–straight (second presentation) direction conditions for the eyes and mosaics are shown.

Fig 2 depicts the eye and mosaic stimuli. The eye stimuli were prepared from color photographs of the full-face neutral expressions of seven females and seven males who were looking either to the left or straight ahead. Only the eyes were used from the photographs, and no other facial features or eyebrows were visible in the stimuli. Mirror images of these stimuli were created using Photoshop 6.0 (Adobe). Eyes looking to the left or right were used for the averted-direction condition and eyes looking straight ahead were used for the straight-direction condition. The mean luminance of the images was kept constant using MATLAB 6.5 (MathWorks).

The mosaic stimuli were constructed from the eye stimuli. First, all of the eye stimuli were divided into small squares (10 vertical × 50 horizontal), and all squares were set to the mean luminance of pixels in each square. To construct objects conveying directional information in a manner similar to the eye stimuli, two sets of 49 small squares with the highest luminance were selected and arranged randomly to construct two large diagonally aligned squares. The squares were aligned diagonally because our preliminary experiment indicated that large squares arranged horizontally looked like eyes. The horizontal center of these large squares was comparable to the pupil positions of the eye stimuli. Other small squares were then arranged randomly in other areas. These manipulations resulted in mosaic stimuli equivalent to the corresponding original eye stimuli in terms of overall luminance and directional information, without the incorporation of eye features.

Stimuli with different direction conditions were shown for the first and second stimulus presentations (i.e., averted after straight or straight after averted) to represent directional changes.

### Procedure

The presentation of stimuli was controlled by SuperLab Pro 2.0 (Cedrus) and implemented using a Windows computer (FSA600, Teknos). Stimuli were presented on a 19-inch CRT monitor (GDM-F400, Sony) at a refresh rate of 100 Hz and a resolution of 1,024 × 768 pixels. The participants’ responses were recorded using a response box (RB-400, Cedrus).

The experiments were conducted individually in a quiet room. The participants were seated comfortably with their heads supported by a chin-and-forehead rest positioned 0.57 m from the monitor. The resulting visual angle subtended by the stimulus was 1.5° vertically × 7.5° horizontally.

Each stimulus was presented three times. In addition, a red cross was presented as the target in 15 trials. Thus, each participant performed a total of 183 trials: 42 trials each of averted eyes-straight eyes, straight eyes-averted eyes, averted mosaics-straight mosaics, and straight mosaics-averted mosaics, as well as 15 target trials. The stimuli were presented in a random order. In each trial, after the presentation of a cross-shaped fixation point for 500 ms, the first stimulus was presented for 500 ms in the center of the visual field. The second stimulus was then presented for 1,000 ms. In each target trial, instead of eyes or mosaic stimuli, the red cross was presented until a response was made. The participants were instructed to press a button using their right forefinger as quickly as possible after detecting the red cross. This task ensured that participants kept their attention on the stimuli, and it prevented the explicit processing of eye gaze. Performance on the target detection was perfect (correct identification rate = 100.0%), with no delay in reaction times (mean ± *SD* = 261.0 ± 15.6 ms). The post-hoc debriefing confirmed that the participants were not aware that the purpose of the experiment was to investigate gaze processing. The participants were also instructed not to blink while the stimuli were being presented. Inter-trial intervals varied randomly between 2,000 and 5,000 ms. To avoid habituation and drowsiness, participants were given short breaks every 45 trials. Prior to data collection, the participants were familiarized with the procedure by performing a block of 10 training trials.

### Data recording

To examine cortical activity, intracranial EEG data were recorded using subdural platinum electrodes (2.3 mm diameter; Ad-tech). Depth platinum electrodes (0.8 mm diameter; Unique Medical) were also inserted to record subcortical activity (data not shown). Electrodes were referenced to electrodes (2.3 mm diameter; Ad-tech) that were embedded inside the scalp of the midline dorsal frontal region. Impedances were balanced and maintained below 5 kΩ. Data were amplified, filtered online (band pass: 0.5–300 Hz), and sampled at 1,000 Hz onto the hard disk drive of the EEG system (EEG-1100; Nihon Kohden). Online monitoring was conducted using a more restricted bandwidth of 0.5–120 Hz. Vertical and horizontal electrooculograms (EOGs) were simultaneously recorded using Ag/AgCl electrodes (Nihon Kohden). As in previous studies [46], off-line visual inspection confirmed that no contamination of the intracranial EEG data by EOGs occurred. Unobtrusive video recording of events was performed using the built-in video camera of the EEG system and an off-line analysis of the videos confirmed that all participants were fully engaged in the tasks.

### Data analysis: Preprocessing

All preprocessing, time–frequency SPM analyses, and ERP analyses were performed using SPM8 (http://www.fil.ion.ucl.ac.uk/spm) implemented in MATLAB R2012b (MathWorks).

Data obtained over 3,000 ms were sampled for each trial; pre-stimulus baseline data were collected for 1,000 ms, and experimental data were collected for 2,000 ms after stimulus onset at a sampling rate of 1,000 Hz. Epochs containing signals with amplitudes > ± 800 μV were excluded from the analyses and any epochs with absolute signal amplitude values > 5 *SD* from the mean or median signal amplitude for each electrode for each participant were rejected as artifacts. The frequencies of artifact-contaminated trials did not significantly differ across the conditions (mean ± *SD* = 6.20 ± 1.84%; *p* > 0.1, repeated-measures analysis of variance).

### Data analysis: Time–frequency SPM analysis

Time–frequency SPM analyses [40,47,48] were performed to assess temporal pole activity. Time-frequency (power) maps were first calculated for each trial using continuous wavelet decomposition with 7-cycle Morlet wavelets during the whole epoch (-1,000–2,000 ms) and from 4 to 300 Hz, which covered theta (4–8 Hz), alpha (8–12 Hz), beta (12–30 Hz), and gamma (30–300 Hz) bands. The time-frequency maps were then cropped to -200–500 ms within the time period evaluated to prevent edge effects of the wavelet transformation. Finally, the time-frequency maps were log-transformed and baseline-corrected separately for each frequency with respect to the mean power over the 200 ms pre-stimulus period.

The time–frequency maps were then converted into two-dimensional images and entered into a general linear model (GLM) based on a fixed-effects analysis of the pooled error from all trials for all participants. Separate analyses were conducted for the first and second stimulus presentations, to test the effects of presence of eyes and changes in eye gaze direction, respectively. We set up a full factorial model including stimulus type (eyes or mosaics), stimulus direction (averted or straight), and hemisphere (right or left) as factors of interest. Corrections for non-sphericity (dependence and possible uneven variance between factor levels) were applied to ensure the assumption of an independent and identically distributed error for the GLM using the restricted maximum likelihood procedure [49]. The window of interest was restricted to the whole frequency range (4–300 Hz) during the post-stimulus period (0–500 ms) using explicit masking. Finally, time-frequency SPM{*T*} data were calculated for each contrast.

Based on our interests, we analyzed the main effects of stimulus type (eyes versus mosaics) and interactions related to the stimulus type factor (i.e., the interactions of stimulus type × stimulus direction, stimulus type × hemisphere, and stimulus type × stimulus direction × hemisphere). For significant interactions, follow-up analyses for simple main effects of stimulus type were conducted. Significantly activated time-frequency clusters were identified if they reached an extent threshold of *p* < 0.05, which was family-wise error corrected for multiple comparisons over the whole time-frequency space (0–500 ms and 4–300 Hz), with a height threshold of *p* < 0.001 (uncorrected).

### Data analysis: ERP

To analyze ERP, one-dimensional SPM analysis [50,51], a variant of the three-dimensional sensor-space-time SPM approach [47,48], was utilized. Because this study focused on a single electrode, the three-dimensional sensor-space-time SPM was reduced to the single-sensor-time SPM. Single trial responses from all trials for all participants were converted into one-dimensional line images after baseline correction for the -200–0 ms time period. The line images were then entered into the GLM in the same manner as for the time–frequency SPM analysis. Planned contrasts and statistical inferences were also performed in the same manner as for the time–frequency SPM analysis.

## Results

### Time–frequency SPM

The time–frequency maps of temporal pole activity were analyzed using the GLM, which included the effects of stimulus type, stimulus direction, and hemisphere (Table 1).

**Table 1:** Time-frequency regions showing significant temporal pole activity.

Contrast	Activation profile					
	Peak			Extent		
	Time	Frequency	*T*-value	Time	Frequency	Cluster size
	(ms)	(Hz)		(ms)	(Hz)	(ms × Hz)
The presence of eyes (first stimulus presentation)						
Main effect of stimulus type^a^	226	121	5.36	215–236	101–150	705
	267	55	3.95	248–282	52–63	249
Interaction of stimulus type × stimulus direction	none					
Interaction of stimulus type × hemisphere^b^	216	97	4.26	209–235	85–102	281
Interaction of stimulus type × stimulus direction × hemisphere	none					
Changes in eye gaze direction (second stimulus presentation)						
Main effect of stimulus type^a^	459	222	4.29	457–464	205–246	205
Interaction of stimulus type × stimulus direction	none					
Interaction of stimulus type × hemisphere^c^	210	47	3.67	197–228	43–51	170
Interaction of stimulus type × stimulus direction × hemisphere	none					

*p* < 0.05 cluster-level family-wise-error corrected.

^a^ contrast: (eyes—mosaics).

^b^ contrast: {(left eyes—left mosaics)—(right eyes—right mosaics)} inclusively masked by positive main effect of stimulus type.

^c^ contrast: {(right eyes—right mosaics)—(left eyes—left mosaics)} inclusively masked by positive main effect of stimulus type.

**Fig 3:**
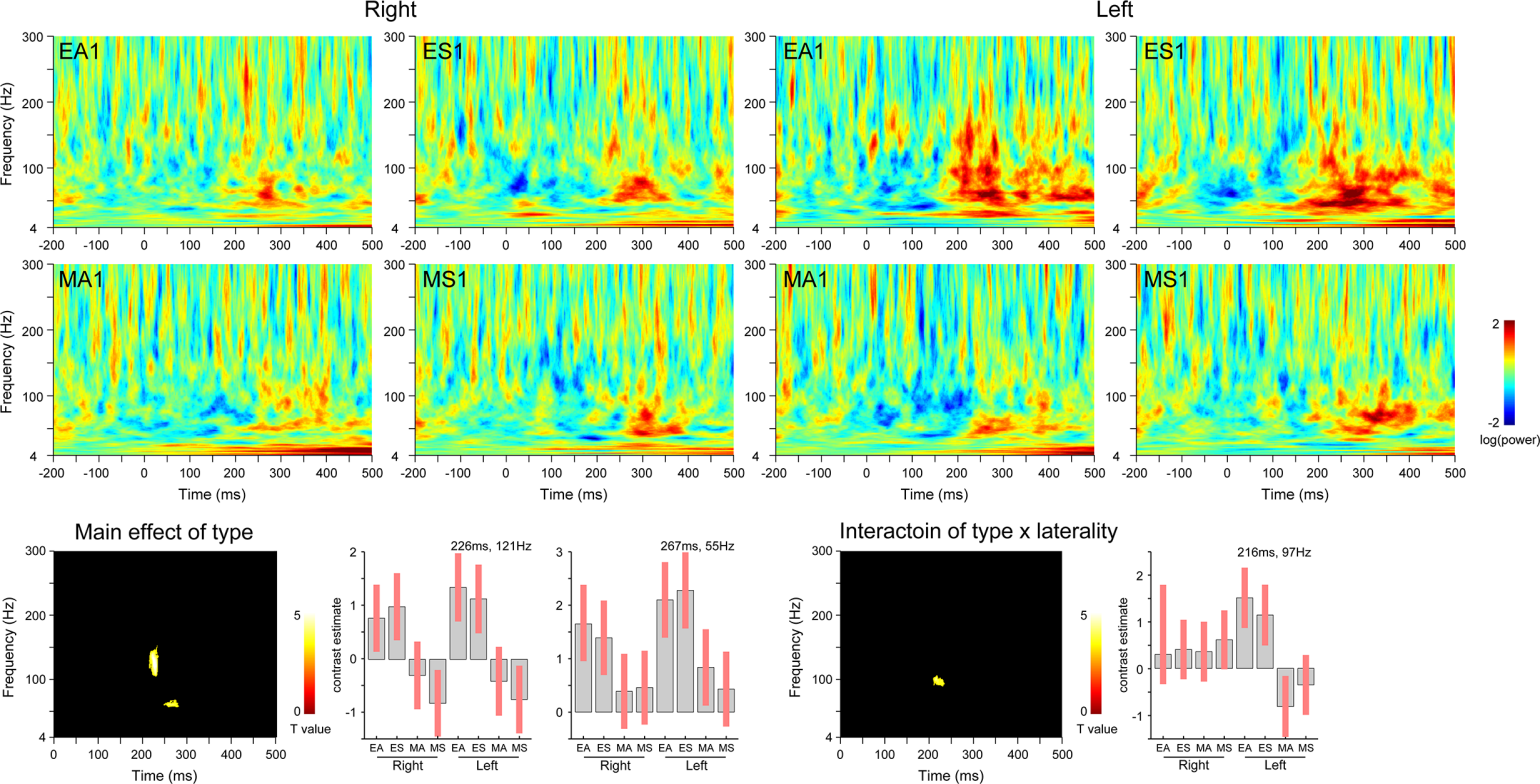
Temporal pole activity in response to the presence of eyes (first stimulus presentation). Upper) Time–frequency maps. Lower) Statistical parametric maps (left) and effect sizes at the peak activation foci (right). *p* < 0.05 cluster-level family-wise error-corrected. EA = averted eyes; ES = straight eyes; MA = averted mosaics; MS = straight mosaics; 1 = first stimulus presentation.

First, temporal pole activity during the first stimulus presentation was analyzed to determine the effect of presence of eyes (Fig 3). The contrast of the main effect of stimulus type (eyes versus mosaics) revealed significant activation in the ranges of 215–236 ms and 101–150 Hz, which indicated that gamma-band activation in the bilateral temporal poles in response to eyes versus mosaics began at 215 ms. There was no significant activation for the interaction of stimulus type × stimulus direction, indicating that the above component was not specific to any gaze direction. The interaction of stimulus type × hemisphere revealed significant activation in the ranges of 209–235 ms and 85–102 Hz. Follow-up analyses indicated that this gamma-band component at a slightly lower frequency range than that of the main effect of stimulus type was evident only in the left temporal pole. There was no significant three-way interaction.

**Fig 4:**
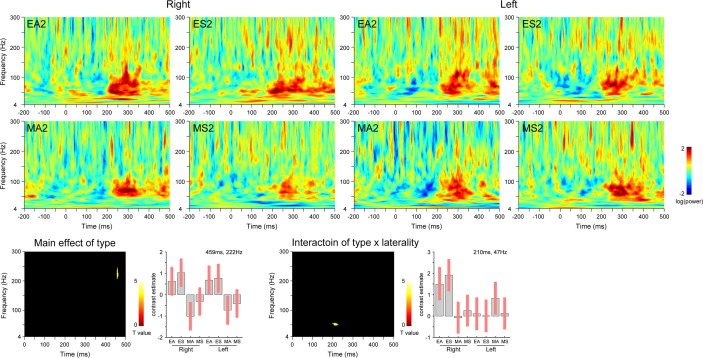
Temporal pole activity in response to changes in eye gaze direction (second stimulus presentation). Upper) Time–frequency maps. Lower) Statistical parametric maps (left) and effect sizes at the peak activation foci (right). *p* < 0.05 cluster-level family-wise error-corrected. EA = averted eyes; ES = straight eyes; MA = averted mosaics; MS = straight mosaics; 2 = second stimulus presentation.

Next, to determine the effect of changes in eye gaze direction, temporal pole activity during the second stimulus presentation was analyzed (Fig 4). The main effect of stimulus type (eyes versus mosaics) was significant at the activation in the ranges of 457–464 ms and 205–246 Hz, which indicated that there was higher gamma-band activation in the bilateral temporal poles in response to eyes versus mosaics. There was no significant activation for the interaction of stimulus type × stimulus direction. The interaction of stimulus type × hemisphere revealed significant activation in the ranges of 197–228 ms and 43–51 Hz. Follow-up analyses revealed that this gamma-band component was evident only in the right temporal pole. There was no significant three-way interaction.

### ERP

**Fig 5:**
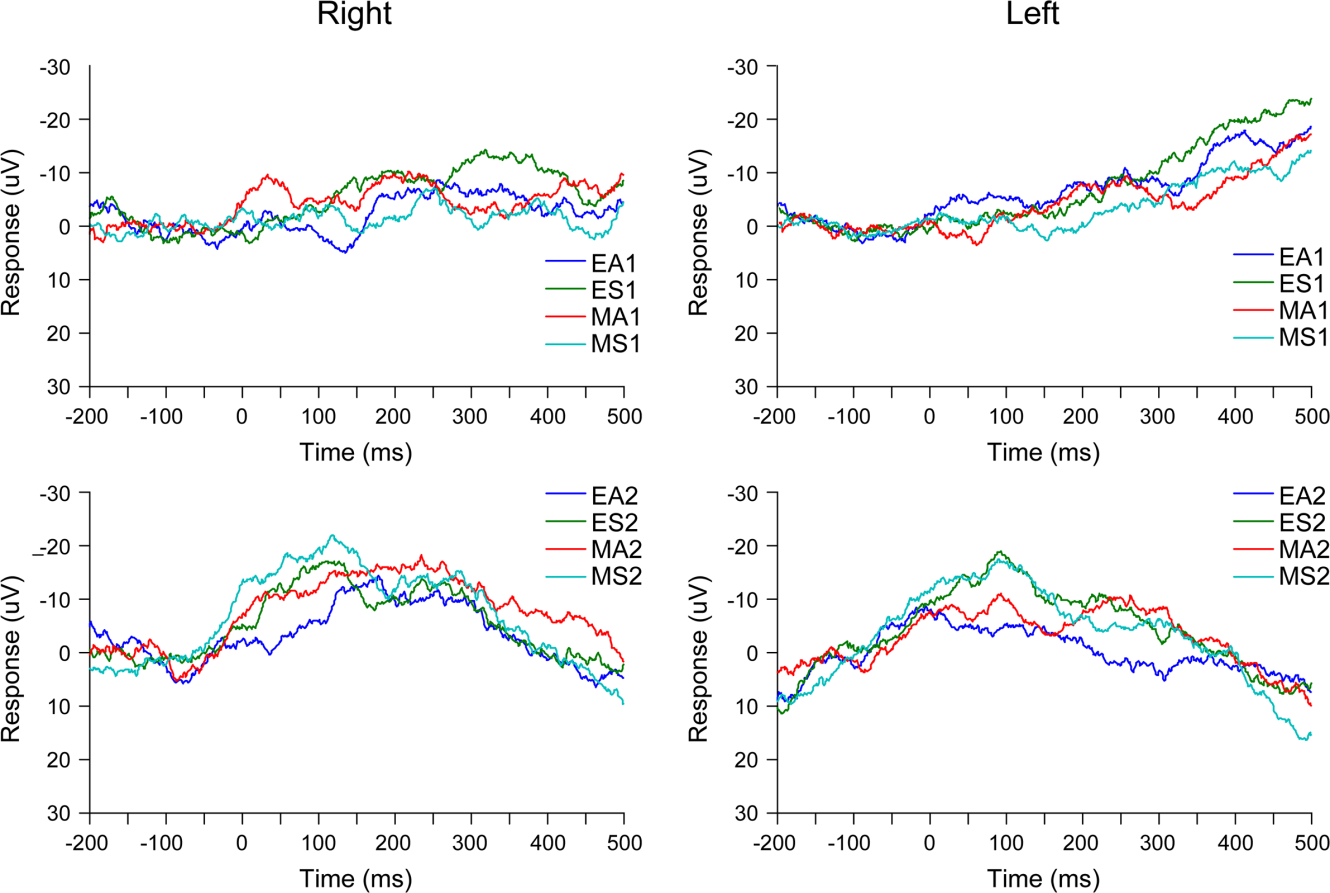
Grand-average event-related potentials in the temporal pole. EA = averted eyes; ES = straight eyes; MA = averted mosaics; MS = straight mosaics; 1 = first stimulus presentation; 2 = second stimulus presentation.

The ERP for each stimulus presentation was analyzed using the GLM in the same manner as the above time-frequency SPM analysis (Fig 5). The results showed no significant activation related to either the main effect of stimulus type or the interactions related to stimulus type.

## Discussion

The time–frequency analysis in the present study demonstrated that there was electric activation in the temporal pole in response to the presence of eyes versus mosaics and to changes in eye gaze direction versus mosaic direction. These results are inconsistent with those of several previous neuroimaging studies that did not observe temporal pole activation in response to the presence of eyes or changes in eye gaze direction (e.g., [10]). However, the discrepant findings may be accounted for by methodological differences. Whereas almost all of the previous studies assessed fMRI data, the present study analyzed electrical signals from the temporal pole. The temporal pole can show weak fMRI signals due to air-tissue inhomogeneity artifacts [52]. The previous studies may also have utilized ill-defined criteria for the temporal pole [31]. On the other hand, the present results are consistent with other neuroimaging studies showing that the temporal pole exhibits activation induced by eye information during active mind reading [32] and that there is a reduction in activation in response to adapted gaze direction [33]. However, these studies did not directly investigate neural activation in response to eyes or eye gaze direction changes. Thus, our results extend those of previous neuroimaging studies and provide the first electrophysiological evidence that the temporal pole is active in response to eyes and eye gaze direction changes.

Furthermore, the time–frequency analysis in the present study provided temporal and frequency profiles for temporal pole activation in response to the presence of eyes and changes in eye gaze direction; activation was identified in the gamma-band beginning at approximately 200 ms. This temporal profile is reasonable when considering the anatomical fact that the temporal pole is located at the endpoint of the occipito–temporal visual stream [31] and the intracranial EEG findings that the inferior occipital gyrus (Sato et al., submitted) and STS region [17] exhibit gamma-band activity that begins at approximately 100 ms in response to eye information and that the temporal pole shows ERP peaks in response to faces about 100–200 ms later than the posterior cortices [7]. Similarly, the frequency profile observed in the present study is consistent with the findings of previous intracranial EEG studies showing that gamma-band activities in several brain regions, including the inferior occipital gyrus (Sato et al., submitted), STS region [17], and amygdala [43], are involved in the processing of eye information. Taken together, the present findings extend the current understanding of neural processing of eye information and indicate that the temporal pole exhibits gamma oscillations beginning at around 200 ms, which is after the activation in the posterior cortices, in response to eyes and eye gaze direction changes.

Irrespective of the commonalities that can be seen in the frequency profiles of the temporal pole and amygdala during the processing of eye information, the temporal profiles and activation patterns of these two regions differ. Although the present results revealed that the temporal pole showed activation in response to the presence of eyes beginning at 215 ms, activation in the amygdala in response to the presence of eyes begins at 170 ms [43]. Whereas the temporal pole showed evident activation in response to both the presence of eyes and changes in eye gaze direction in the present study, the amygdala only shows clear activation in response to the presence of eyes [43]. These data indicate that the temporal pole conducts different types of processing of eye information at a later time point relative to the amygdala. Because the temporal pole and the amygdala have bidirectional connections [26], the temporal pole may receive inputs from the amygdala along with inputs from the posterior visual cortices. In contrast, the amygdala appears to conduct the processing of eyes prior to the receipt of visual inputs from the temporal pole using different pathways, such as the subcortical pathway via the superior colliculus and pulvinar [53,54] or the cortical pathway from the posterior regions [55,56].

The present results also revealed functional hemispheric differences such that the left and right temporal poles showed more evident gamma-band activation in response to the presence of eyes and changes in eye gaze direction, respectively. The existence of hemispheric asymmetry in the temporal pole is consistent with findings from other literatures, such as semantic cognition (for a review, see [57]). The left hemispheric dominance for processing eyes may be in line with a study of brain-damaged patients that observed a left hemispheric dominance in mind reading ability using information from eyes [58]. The right hemispheric dominance regarding eye gaze direction changes is consistent with several previous neuroimaging studies showing a right hemispheric dominance during the processing of dynamic facial signals (e.g., [59]). However, it must be noted that the electrode placement in the present study was based on anatomical information, and hence, it is possible that the electrodes in the right and left temporal poles were not comparable functionally. Further investigation is necessary to determine whether a functional hemispheric asymmetry exists with respect to temporal pole activities during the processing of eye information.

In contrast to the hypotheses of the present study, the results did not reveal evident ERP or low-frequency band activation in response to the presence of eyes and changes in eye gaze direction. Although drawing of any conclusions based on a null finding should be postponed, this result suggests that the temporal pole may not process eye information using low-frequency activity. The results showing that the gamma-band activity exhibited eye information-related activity, but low-frequency band activity did not, may also have some interesting implications. It has been proposed that the brain generally uses electrical activity in low-frequency bands, such as the theta-band, for long-range inter-regional communication and that this low-frequency activity entrains local intra-regional gamma-band activation that corresponds to the computation of cell populations (e.g., [35,60]). The present findings suggest that this cross-frequency theta–gamma coupling may not be involved in the processing of eye information in the temporal pole. Instead, it may be possible that inter-regional communication between the temporal pole and other brain areas during the processing of eye information is accomplished via the same-frequency gamma–gamma coupling (cf. [61]).

The present findings have several implications. First, the activation of the temporal pole and its specific temporal profile during the processing of eyes and eye gaze direction changes updates the current understanding of the spatiotemporal neural network dynamics involved in the processing of eye information. Although the temporal pole is commonly associated with the processing of social interactions [31], this area has received relatively little attention regarding the processing of eye information [62–64]. Second, the occurrence of temporal pole activation during the processing of eye information suggests that the impaired social functioning observed in monkeys following damage to this region (e.g., [20]) could at least partially be attributed to the impaired processing of eye information. Likewise, human studies have reported that the brain damage [65,66] and atrophy associated with semantic dementia [67,68] including the temporal pole induce social malfunctioning and this may be related to impaired processing of eye information. Finally, the involvement of gamma oscillations in the temporal pole during the processing of eye information corroborates previous evidence showing that the brain uses this frequency range to accomplish information processing (e.g., [69]; for a review, see [70]).

A limitation of this study should be acknowledged. We asked participants to engage in a dummy task to investigate automatic activation in response to eyes and eye gaze direction changes. However, this task did not reveal any details regarding the processing of eye information associated with temporal pole activity. Different tasks might enhance or suppress activity in the temporal pole at particular temporal and frequency points. For example, a previous fMRI study has observed activation in the temporal pole during intentional mind reading based on eye information [32]. This particular mind reading task during the observation of eyes may enhance gamma-band activity beginning at about 200 ms, as we found in the present study, or it may induce additional later gamma-band activation in the temporal pole. In future studies, participants should be asked to engage in intentional cognitive processes in response to eyes or eye gaze direction changes to specify the functional correlates of temporal pole activity.

In summary, the present intracranial EEG data revealed that the bilateral temporal poles exhibited a greater degree of gamma-band activation beginning at 215 ms in response to eyes compared with mosaics, irrespective of gaze direction. Additionally, the right temporal pole showed a greater degree of gamma-band activation beginning at 197 ms in response to directional changes of the eyes compared with mosaics. These results suggest that the temporal pole uses gamma oscillations to process the presence of eyes and changes in eye gaze direction at a relatively late time stage compared with brain regions in the posterior cortices, such as the inferior occipital gyrus and STS region.

## Supporting Information

S1 Dataset Datasets of Temporal pole activity. (XLSX)
